# Performance evaluation in [^18^F]Florbetaben brain PET images classification using 3D Convolutional Neural Network

**DOI:** 10.1371/journal.pone.0258214

**Published:** 2021-10-20

**Authors:** Seung-Yeon Lee, Hyeon Kang, Jong-Hun Jeong, Do-young Kang

**Affiliations:** 1 Department of Translational Biomedical Sciences, Dong-A University, Busan, Korea; 2 Institute of Convergence Bio-Health, Dong-A University, Busan, Korea; 3 DEEPNOID Inc., Seoul, Korea; 4 Department of Nuclear Medicine, Dong-A University Medical Center, Busan, Korea; Fondazione Istituto G.Giglio di Cefalu, ITALY

## Abstract

High accuracy has been reported in deep learning classification for amyloid brain scans, an important factor in Alzheimer’s disease diagnosis. However, the possibility of overfitting should be considered, as this model is fitted with sample data. Therefore, we created and evaluated an [^18^F]Florbetaben amyloid brain positron emission tomography (PET) scan classification model with a Dong-A University Hospital (DAUH) dataset based on a convolutional neural network (CNN), and performed external validation with the Alzheimer’s Disease Neuroimaging Initiative dataset. Spatial normalization, count normalization, and skull stripping preprocessing were performed on the DAUH and external datasets. However, smoothing was only performed on the external dataset. Three types of models were used, depending on their structure: Inception3D, ResNet3D, and VGG3D. After training with 80% of the DAUH dataset, an appropriate model was selected, and the rest of the DAUH dataset was used for model evaluation. The generalization potential of the selected model was then validated using the external dataset. The accuracy of the model evaluation for Inception3D, ResNet3D, and VGG3D was 95.4%, 92.0%, and 97.7%, and the accuracy of the external validation was 76.7%, 67.1%, and 85.3%, respectively. Inception3D and ResNet3D were retrained with the external dataset; then, the area under the curve was compared to determine the binary classification performance with a significance level of less than 0.05. When external validation was performed again after fine tuning, the performance improved to 15.3%p for Inception3D and 16.9%p for ResNet3D. In [^18^F]Florbetaben amyloid brain PET scan classification using CNN, the generalization potential can be seen through external validation. When there is a significant difference between the model classification performance and the external validation, changing the model structure or fine tuning the model can help improve the classification performance, and the optimal model can also be found by collaborating through a web-based open platform.

## 1 Introduction

Alzheimer’s disease (AD) is a progressive and irreversible neurodegenerative disease in which a patient gradually loses memory, mental function, and the ability to continue daily activities [1]. As there is no effective treatment for AD, an accurate diagnosis is essential for developing the patient’s future treatment plan. Neuroimaging technology using AD-related biomarkers is widely used to increase the reliability of AD diagnosis [2]. Radiotracers are among the types of biomarkers that can be injected into the subject’s body and observed via PET. Representative radiotracers used to diagnose AD include 2-[^18^F]fluoro-D-glucose, which can identify the degree of brain metabolism, and [^18^F]Florbetaben, [^18^F]Florbetapir and [^18^F]Flutemetamol which can observe brain amyloid plaque load.

According to specific or diagnostic criteria [3–5], several systems apply brain PET scans to machine learning and deep learning models to train, evaluate, and classify images. Moreover, there is also recent work using them on automatic and semi-automatic segmentation algorithms in PET [6, 7]. In one study, an AD diagnosis classifier using PCA and SVM was utilized after image dimension reduction of [^18^F]Florbetaben brain PET images [8], and in another, images were classified according to amyloid deposition with an accuracy of 89% [9] using Visual Geometry Group (VGG) 16 [10], which is a well-known structure among convolutional neural networks (CNN) [11, 12] that specializes in image feature extraction using deep learning technology.

Meanwhile, because of the nature of medical images, acquisition costs can be high; thus, it is not easy to construct a large dataset. When a model is trained and tested with a limited number of datasets, to confirm the generalization possibility, it is necessary to configure an external dataset that is different from the source of the training dataset and to validate its potential.

Previous studies, such as the external validation of pancreatic cancer from CT images [13] and the external validation of malignancy risk prediction of lung nodules [14], have been reported. It is necessary to apply the validation process to brain imaging to build a model and then to externally validate the model with the same type of brain images obtained from other sites.

In this study, brain PET scans of [^18^F]Florbetaben, a diagnostic radiotracer that visualizes the classification of *β*-amyloid (A*β*), the main component of amyloid plaques found in the brain, were acquired from Dong-A University Hospital (DAUH) and used to construct a dataset. We created models that receive 3D voxel input by deriving characteristic structures from the well-known CNN structures Inception, VGG, and ResNet. We then selected one representative model for each structure after training according to the model selection criteria. With this model, the images acquired from the Alzheimer’s Disease Neuroimaging Initiative (ADNI) were used as a dataset and externally validated to examine the possibility of generalization.

## 2 Materials and methods

### 2.1 Data acquisition

The DAUH dataset, listed in Table 1, comprises a total of 432 subjects according to the visually assessed criteria of [^18^F]Florbetaben PET. An available database was collected from 2015 to 2020 from the Department of Nuclear Medicine at DAUH for a possible population of [^18^F]Florbetaben PET. Labeling for A*β* negative and positive was performed according to the decision of a nuclear medicine specialist at DAUH.

**Table 1:** Demographics and positivity of study participants according to each dataset group.

Dataset	DAUH		External	
n	432		251	
Visual Assessment	Negative	Positive	Negative	Positive
Subjects	191	241	142	109
Age	68.1	69.9	70.8	73.7
Sex, male %	35.6	44.4	40.8	56.9
Education	9.1	10	16.1	16

PET scans of the possible population were acquired using a Biograph 40 mCT Flow PET/CT Scanner (Siemens Healthcare, Knoxville, TN, USA) and reconstructed via UltraHD-PET (TrueX-TOF) to obtain PET images, and the obtained images were processed with a 3mm FWHM Gaussian filter. All patients underwent a 20 min positron emission scan at 90 min after intravenous injection of 300mBq of [^18^F]Florbetaben (NeuraCeq, Piramal, Mumbai, India), and the helical CT scans were acquired with a 0.5 s rotation time at 100 kVp and 228 mAs. The images were finally stored in the DICOM format.

Finally, [^18^F]Florbetaben PET images collected from the Alzheimer’s Neuroimaging Initiative (ADNI) database (www.loni.ucla.edu/ADNI) were used as an external dataset in this study. The database includes scans of subjects with normal control, mild cognitive impairment (MCI), and AD. These scans were preprocessed as an ADNI internal protocol and co-registration, averaging, size changing, standardization, and smoothing processes were performed. However, since ADNI does not determine whether the [^18^F]Florbetaben scans are A*β* negative or positive, classification was performed by the nuclear medicine specialists at DAUH.

**Fig 1:**
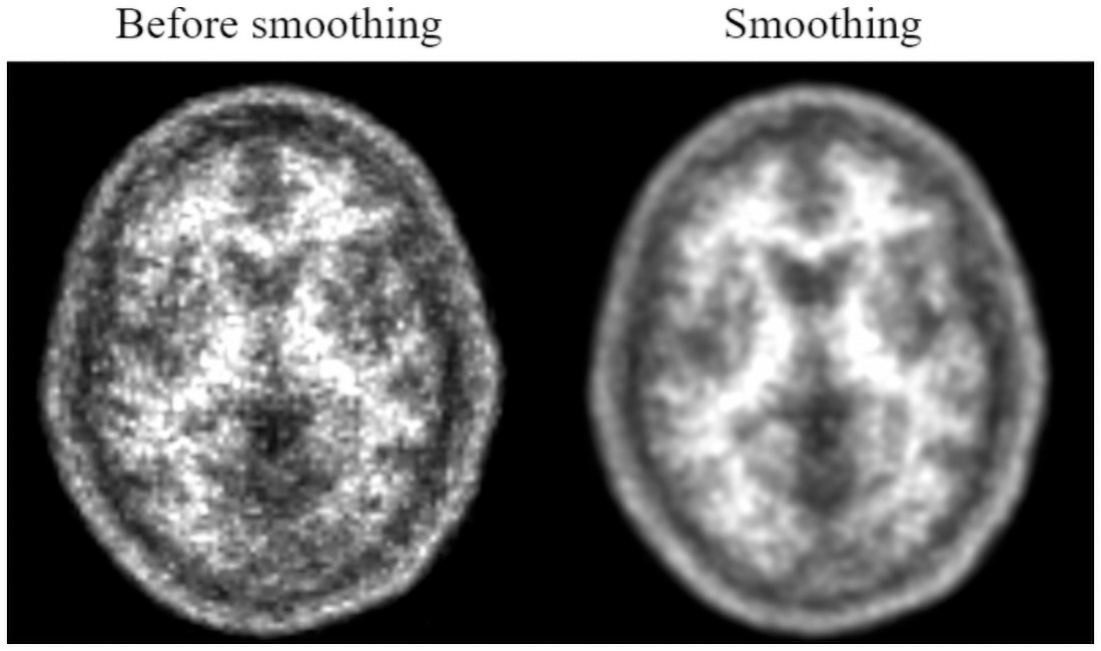
Smoothing of the external data. After the smoothing process, an image in which noise is reduced can be obtained.

Unlike the DAUH dataset, images from the external dataset were not processed with 3 mm FWHM Gaussian filters. To achieve the same conditions, a 3 mm FWHM Gaussian filter was used to process the statistical parametric mapping (SPM) after the acquisition. An example of Gaussian filter processing is shown in Fig 1.

### 2.2 Image preprocessing in common

We performed spatial normalization and count normalization to classify A*β* deposition using brain PET. We used the SPM library [15] based on MATLAB. All dataset groups applied the same preprocessing procedure using the same protocol, even if there were differences in the source and collection times.

Spatial normalization is the registration of the original image to a specific PET template. Subjects with various brain shapes can be compared through image registration by mapping the subject’s PET image to a reference brain template [16].

Meanwhile, we made a reference brain PET template in-house. Co-registration was performed with a PET image and a paired CT image obtained from a subject. The template images are average images of 20 normal brains, 20 Alzheimer’s dementia brains, and 40 left and right inversion images, for a total of 80 images. After image registration, the tissue is stretched or compressed to fit the template brain. We can average the PETs of multiple individuals to reconstruct them into a reference brain space and provide atlas anatomical addresses mapped to the same reference brain space at the data locations in the image. We also performed a cropping process that cuts the empty space around the brain, reducing its size from 400 × 400 × 110 to 95 × 79 × 68.

Count normalization is intended for numerical comparisons between images because the image intensity level varies due to differences in the number of radioactive isotopes administered, individual characteristics, or individual body conditions. This normalization was performed assuming that the absorption of radioactive tracers in a brain region is constant for each person. Count normalization normalizes the entire observation area to the value of the area representing non-specific, lesion-independent absorption, allowing absolute and relative comparisons in specific absorption areas of the patient-patient image [17].

The [^18^F]Florbetaben radiotracer exhibits non-specific uptake in the cerebellar region and specific uptake in the gray matter region of the cerebrum; thus, count normalization was performed by using the cerebellar region of the PET template applied in spatial normalization to each patient image.

In addition, the skull, a non-brain tissue, is included in the image because it is spatially normalized with a CT-driven PET template. The presence of these non-brain tissues is considered an obstacle in brain image analysis. Therefore, in brain imaging analysis studies, a preprocessing commonly referred to as skull stripping is required [18].

**Fig 2:**
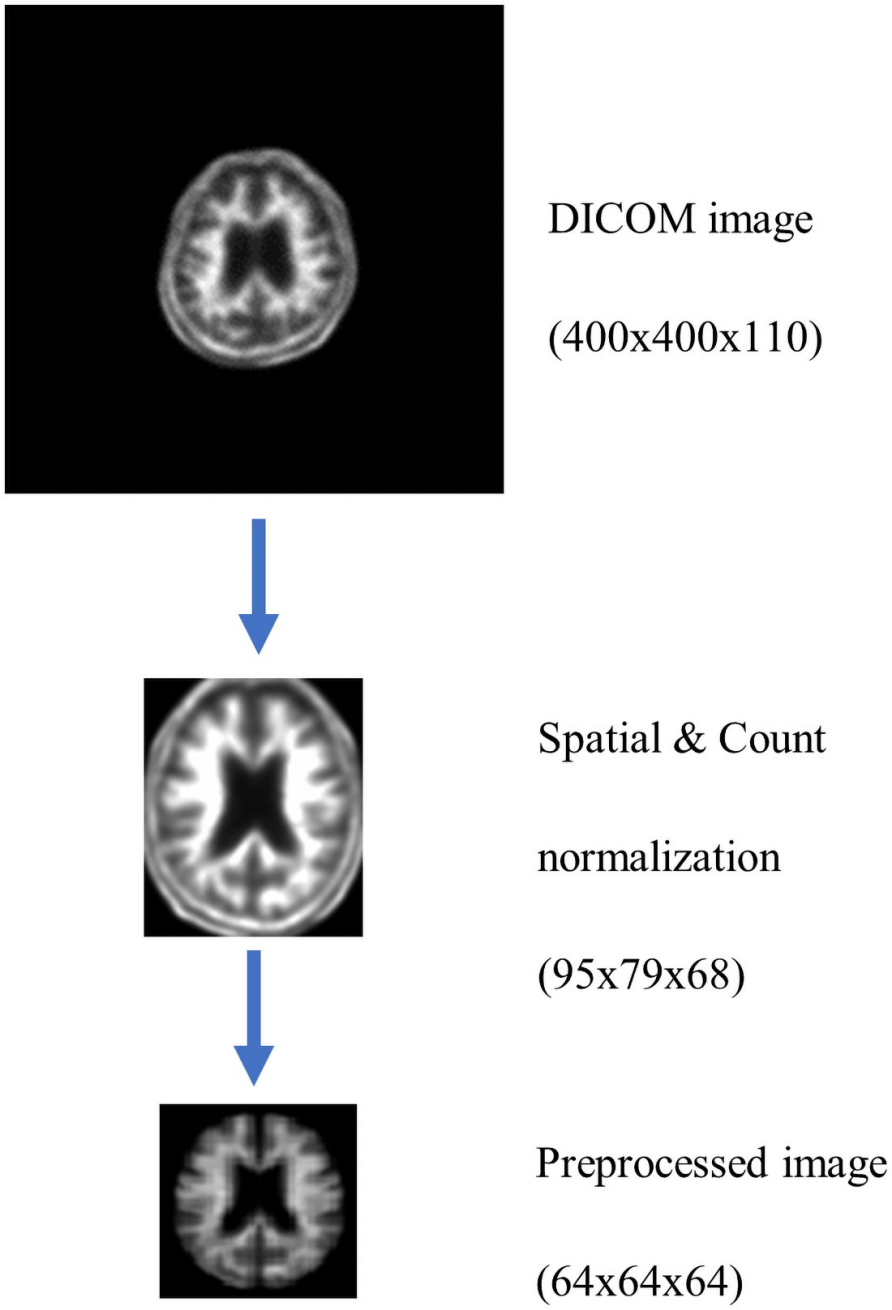
Commonly applied preprocessing.

In the [^18^F]Florbetaben amyloid brain PET classification model, spatial normalization, count normalization, and skull stripping are commonly performed for all datasets, and an example of a brain PET scan image that has been preprocessed is shown in Fig 2.

### 2.3 Model architecture

**Fig 3:**
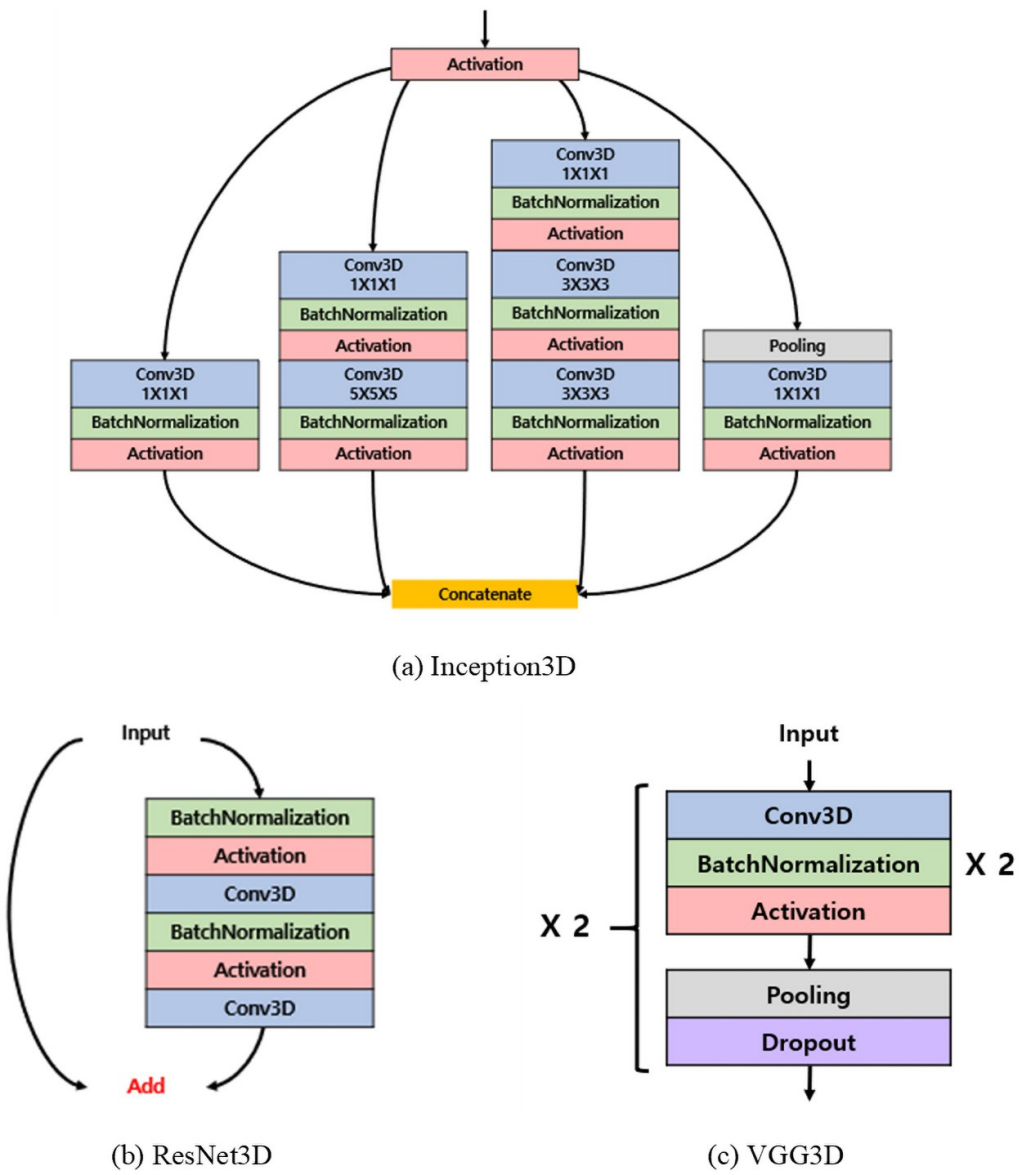
Primary structure of the models. (a) and (b) are the most characteristic parts of the structure, and (c) is the overall structure of the model. The ‘×2’ written on the side means that the process is repeated as many times as the number.

In this study, we apply CNNs of three well-known architectures to the amyloid classification problem in [^18^F]Florbetaben brain PET. The architectures considered are Inception [19, 20], ResNet [21], and VGG19 [10]. The reasons for choosing these models were to achieve the best performance in various tasks, use a small kernel (3 × 3) [10] instead of a large kernel, utilize a deep but sparse network structure [19], and provide residual connectivity [21]. We implemented the model ourselves by adopting parts of the primary characteristics of these structures and applying 3D convolution filter. Each representative structure is shown in Fig 3.

To briefly summarize the meaning of each layer:

- Conv3D: 3D convolution kernel layer.
- BatchNormalization: To avoid gradient vanishing problems due to structural complexity and speed up the training [22].
- Activation: When the data that has passed through the layer is transmitted to the next layer, it plays a role in determining whether to transmit the input data according to a specific criterion, and all model structures in this study apply the Rectifier Linear Unit (ReLU) [23] activation function.
- Concatenate: To connect data horizontally.
- Pooling: Also known as sub-sampling, the image is reduced by selecting a large value or taking an average in the corresponding receptive field.
- Dropout: Only certain weights are kept at a certain probability and the remaining connected units are diluted. This is known to prevent network overfitting [24].

Recently, 3D CNN research has been actively conducted to extract features of 3D medical images for classification [25]. Moreover, because brain images are volume data, 3D CNNs can be configured for image classification to extract 3D spatial features from 3D PET images. Accordingly, Inception, ResNet, and VGG models are constructed in the form of a 3D CNN and are named Inception3D, ResNet3D, and VGG3D in our experiment.

### 2.4 Model selection and evaluation

**Fig 4:**
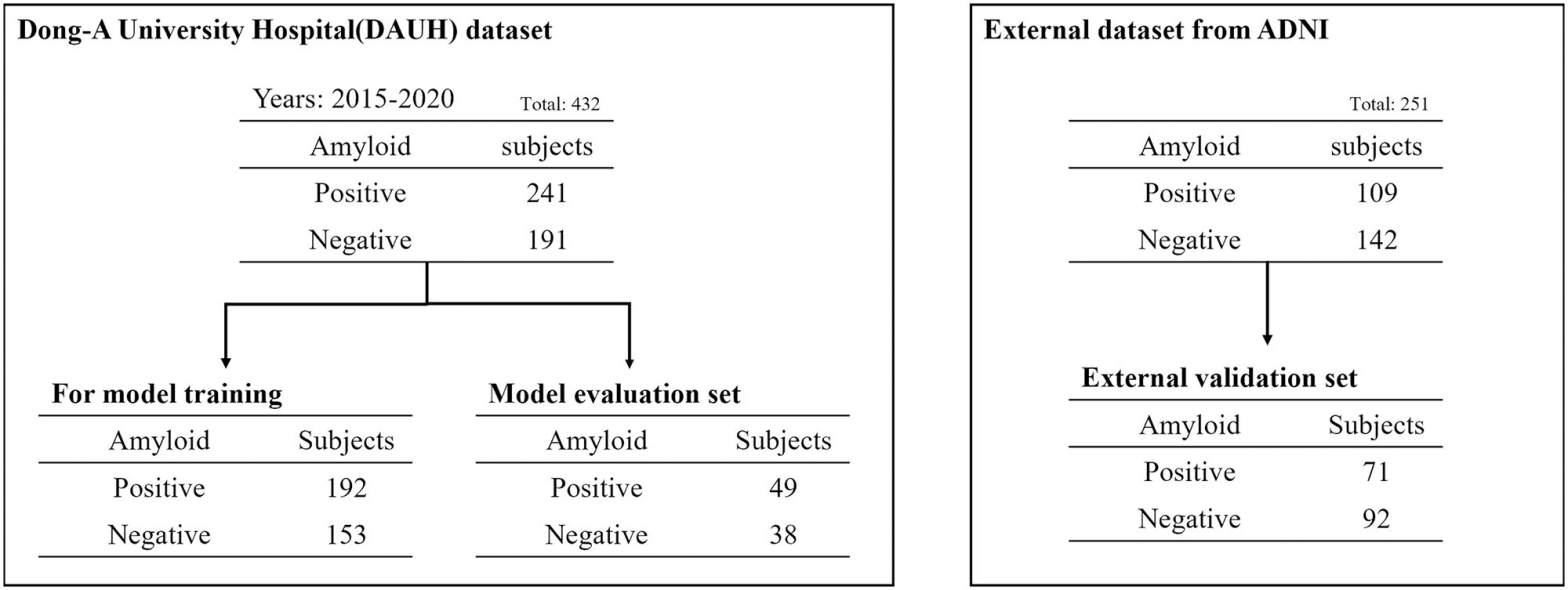
DAUH and external datasets for model training and validation.

**Fig 5:**
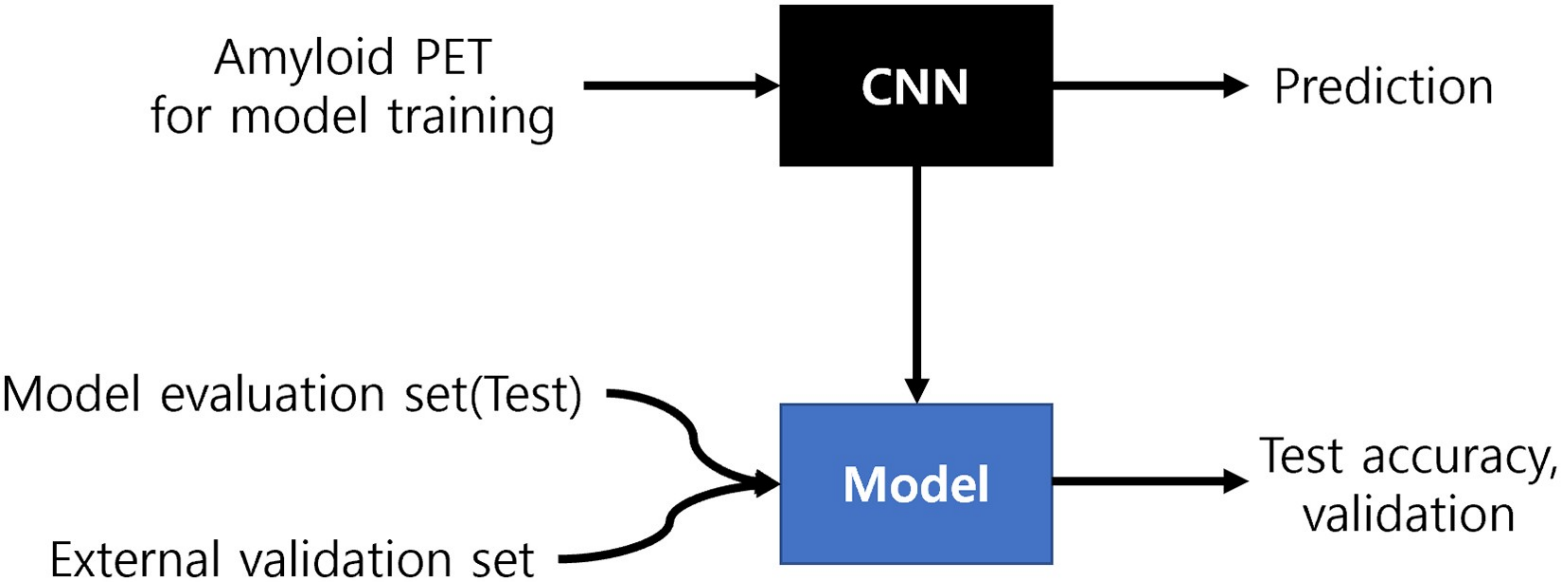
Overall workflow.

Eighty percent of the DAUH dataset, as shown in Fig 4, was used for model training and the rest is used for model evaluation. The samples were splitted with Stratified ShuffleSplit cross-validator (https://scikit-learn.org/stable/modules/generated/sklearn.model_selection.StratifiedShuffleSplit.html), which was randomly extracted from each group labeled amyloid positive and negative. Along with the training set, there was also a validation set used to ensure that the model is trained well. This is explained in more detail in Fig 5.

The model training was conducted by four-fold cross-validation with 192 amyloid positive subjects and 153 amyloid negative subjects from the DAUH dataset. If the dataset is small, the reliability of the performance evaluation is reduced. If the performance varies depending on how the validation set is held, the effect of the match will bias the model evaluation performance. To solve this, cross-validation ensures that all data are used as a validation set at least once.

**Fig 6:**
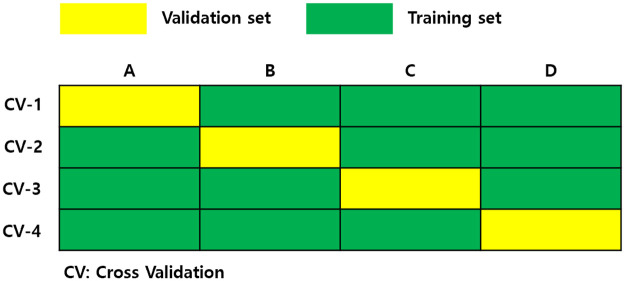
Four-fold cross-validation for each iteration.

As shown in Fig 6, the entire dataset can be divided into four subsets; the first subset is used as the validation set in the first iteration, and the remaining subsets are used as the training set. In the second iteration, the second subset is used as the validation set, and the remaining subsets are used as the training set. By repeating the number of subsets in this manner, we could select the lowest loss model out of the four performances.

All models of each structure were selected through four-fold cross-validation [26] and evaluated with the model evaluation set of the DAUH dataset specified in Fig 4.

In the external dataset, some samples were randomly extracted using the same sample extraction method. If the accuracy was significantly different between the model evaluation and the external validation, it is possible that a part of the external dataset could be included in the training set to acquire generalization performance. In that case, only a portion of the dataset was composed of the external validation set.

## 3 Results

We investigated information about the model and the time required for each experiment. Specifications about the models can be viewed in Table 2. Experiment time consists of training, image loading, and prediction using the validation set. The required time for each network is Inception3D 1.46hrs, ResNet3D 1.9hours, and VGG3D 1.90 hours. Inference time took about 0.24 seconds per subject, and all experiments were performed on a workstation with four NVIDIA Titan Xp GPUs.

**Table 2:** Specifications about the models.

	Inception3D	ResNet3D	VGG3D
Total params	6,098,530	17,620,196	17,236,386
Trainable params	6,093,538	17,612,642	17,236,002
Non-trainable params	4,992	7,554	384
Size on disk	70	202	195

### 3.1 Data distribution

**Fig 7:**
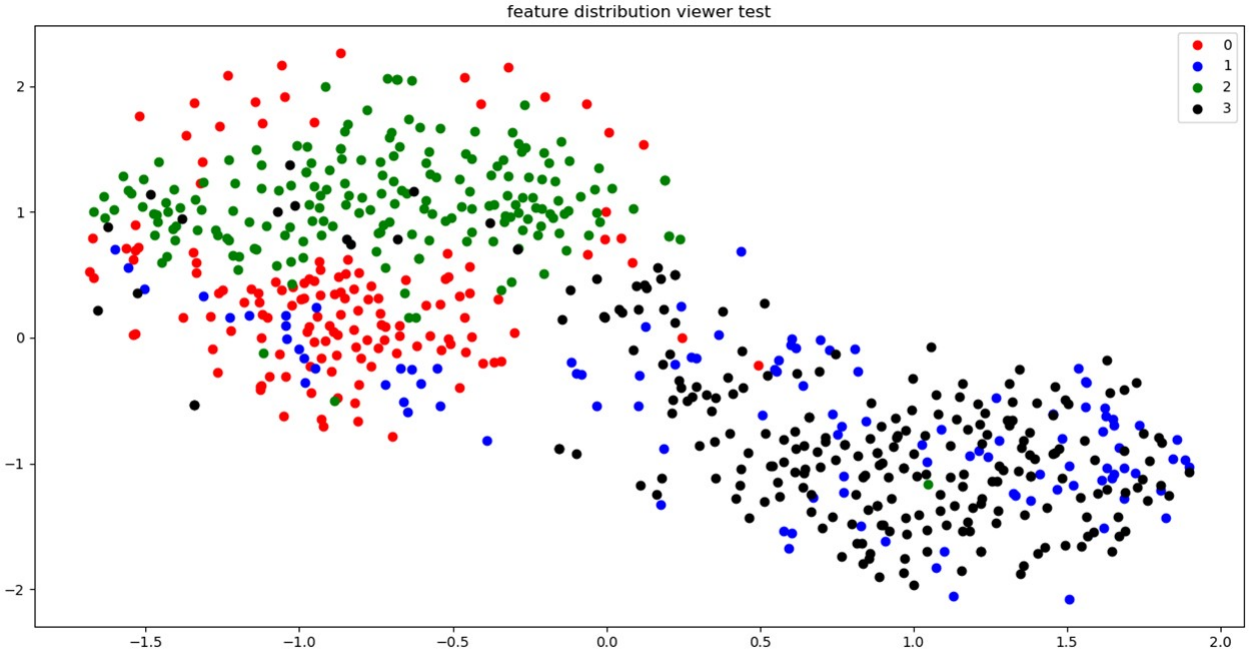
Dataset distribution using t-SNE visualization.

To confirm the data distribution of the preprocessed DAUH and external datasets before CNN-based analysis, as shown in Fig 7, t-SNE (Stochastic Neighbor Embedding) [27] visualization, which is widely used for visualization after a data dimension reduction, was performed. Although the sources of the datasets are different, the same groups have a similar distribution. In other words, data similarity was observed between the same groups.

### 3.2 Model evaluation

The model evaluation results of the Inception3D, ResNet3D, and VGG3D models after training the models with the DAUH training dataset are summarized in Table 3. To evaluate the performance of each model, three metrics were considered: sensitivity, specificity, and accuracy.

**Table 3:** Classification evaluation metrics.

	Inception3D		ResNet3D		VGG3D	
	Model Evaluation	External Validation	Model Evaluation	External Validation	Model Evaluation	External Validation
AUC	0.996	0.883	0.968	0.901	0.98	0.945
Sensitivity	0.918	0.845	0.918	0.944	0.959	0.831
Specificity	1	0.707	0.921	0.469	1	0.945
PPV	1	0.69	0.938	0.458	1	0.831
NPV	0.905	0.855	0.897	0.918	0.95	0.87
Accuracy	0.954	0.767	0.92	0.671	0.977	0.87
Standard Deviation	0.487	0.452	0.482	0.351	0.95	0.853
95% Confidence Interval	0.950 to 1.000	0.823 to 0.928	0.907 to 0.994	0.845 to 0.942	0.923 to 0.998	0.898 to 0.974

Sensitivity is the proportion of subjects who are inferred to be positive among all A*β*-positive subjects and is defined as follows:

$$\begin{array}{c} Sensitivity=\frac{TP}{TP+FN} \end{array}$$

where TP represents the number of true positives and FN represents the number of false negatives.

Specificity is the proportion of subjects who are inferred to be negative among all A*β*-negative subjects and is defined as follows:

$$\begin{array}{c} Specificity=\frac{TN}{TN+FP} \end{array}$$

where TN represents the number of true negatives and FP indicates the number of false positives.

PPV(Positive Predictive Value) is the probability that those that come out A*β* positive actually have A*β* positive according to the ground truth. NPV(Negative Predictive Value) is the probability that those that come out A*β* negative actually have A*β* negative according to the ground truth.

$$\begin{array}{c} PPV=\frac{TP}{TP+FP} \end{array}$$

$$\begin{array}{c} NPV=\frac{TN}{FN+TN} \end{array}$$

Accuracy is the degree of closeness between the predicted value and the actual value of the subjects. It is calculated as the number of true positives and negatives among all accurate and evaluated subjects as follows:

$$\begin{array}{c} Accuracy=\frac{TP+TN}{TP+FN+TN+FP} \end{array}$$

**Fig 8:**
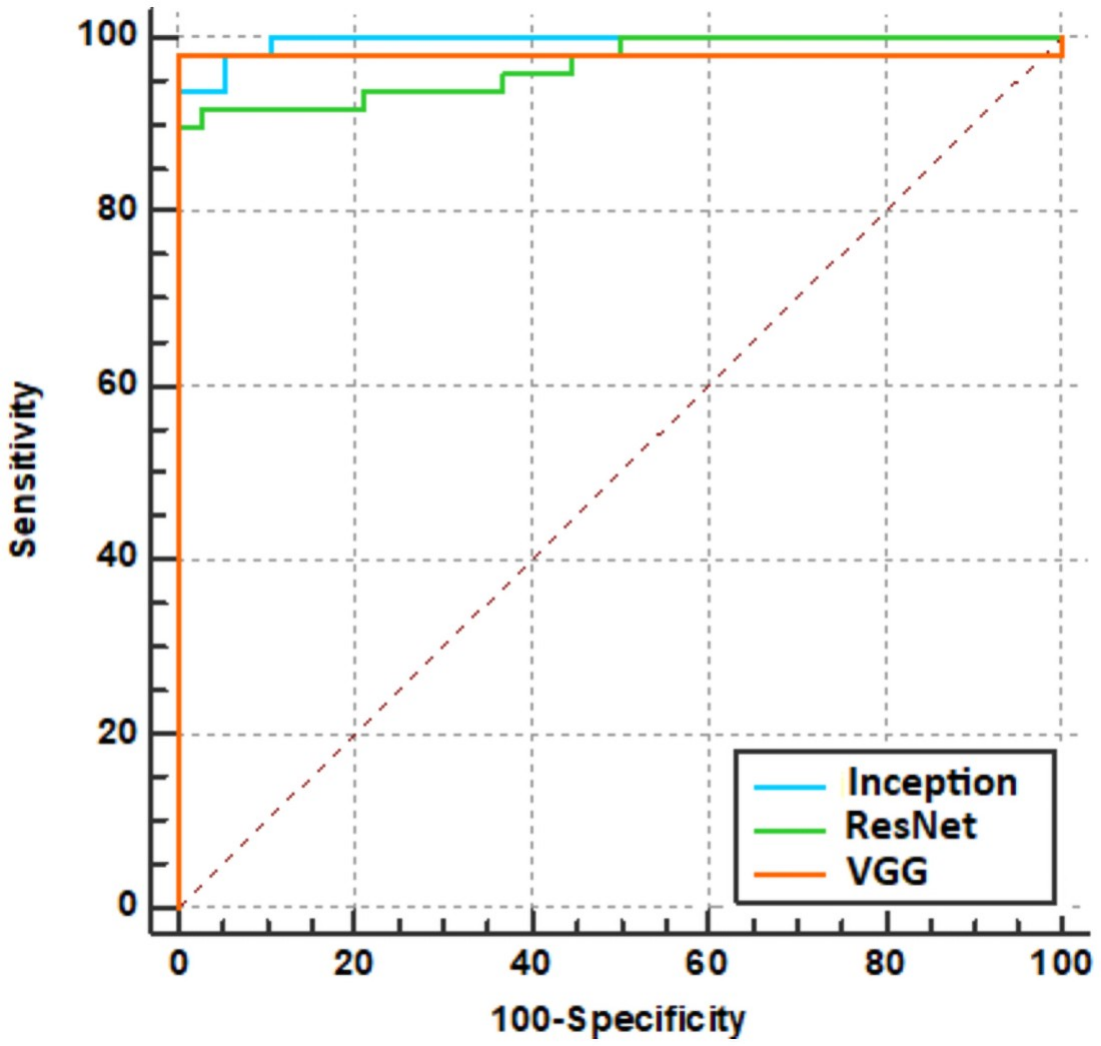
ROC curves for each model evaluation.

We plotted the receiver operating characteristic (ROC) curve of our method for A*β* positivity, as shown in Fig 8, and calculated the area under the curve (AUC). The AUC value was close to 1 for all three models.

Table 4 summarizes the pairwise comparison results of ROC curves obtained from the Inception3D, ResNet3D, and VGG3D inferences that performed model evaluation. At the 95% significance level, since both p-values are greater than 0.05, the corresponding two comparison areas are not significantly different.

**Table 4:** Comparison of ROC curves.

Pairwise comparison of ROC curves for each model evaluation				
	Difference between areas	SE	95% CI	P-value
Inception3D vs. ResNet3D	0.0274	0.0154	-0.00282 to 0.0576	0.0756
Inception3D vs. VGG3D	0.0161	0.0208	-0.0246 to 0.0569	0.4384
ResNet3D vs. VGG3D	0.0113	0.0203	-0.0285 to 0.0510	0.5780
Comparison of independent ROC curves with the model evaluations and the external validations				
Inception3D	0.113	0.0266	0.0610 to 0.165	<0.05
ResNet3D	0.0672	0.0306	0.00733 to 0.127	<0.05
VGG3D	0.0349	0.0269	-0.0179 to 0.0876	0.1950

Note. SE = standard error, CI = confidence interval

### 3.3 External validation

**Fig 9:**
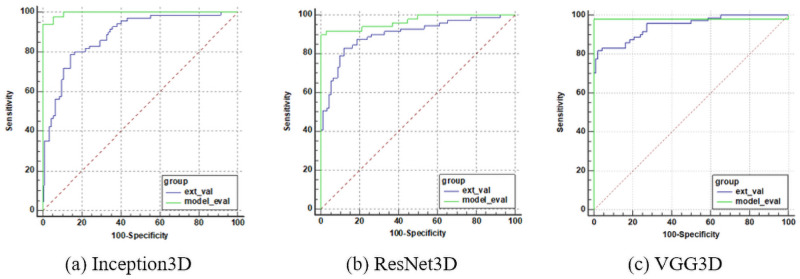
ROC curve comparison for each model. In the legend of each plot, ‘*ext*_*val*’ means the external validation, and ‘*model*_*eval*’ means the model evaluation.

With respect to accuracy and AUC, VGG3D showed the best classification performance. As summarized in Table 3, the AUC of the external validations was lower than that of the model evaluation, and a comparison of the p-values summarized in Table 4 indicates that Inception3D and ResNet3D were below the significance level; thus, there were significant differences in the performance evaluation. However, there was no significant difference between the model evaluation and the external validation for the VGG model, as shown by the significance level, p = 0.1950. The ROC curves of the comparison are plotted in Fig 9.

### 3.4 Retraining model

This is a method of redefining the model to create a generalized model by additionally retraining a part of the external dataset to the model trained with the DAUH training set. In this part, excluding the external validation set shown in Fig 4, the 38 A*β* positives and 50 A*β* negatives were configured as a retraining set for fine tuning. the pre-trained model was imported as it is, and all layers were retrained.

**Fig 10:**
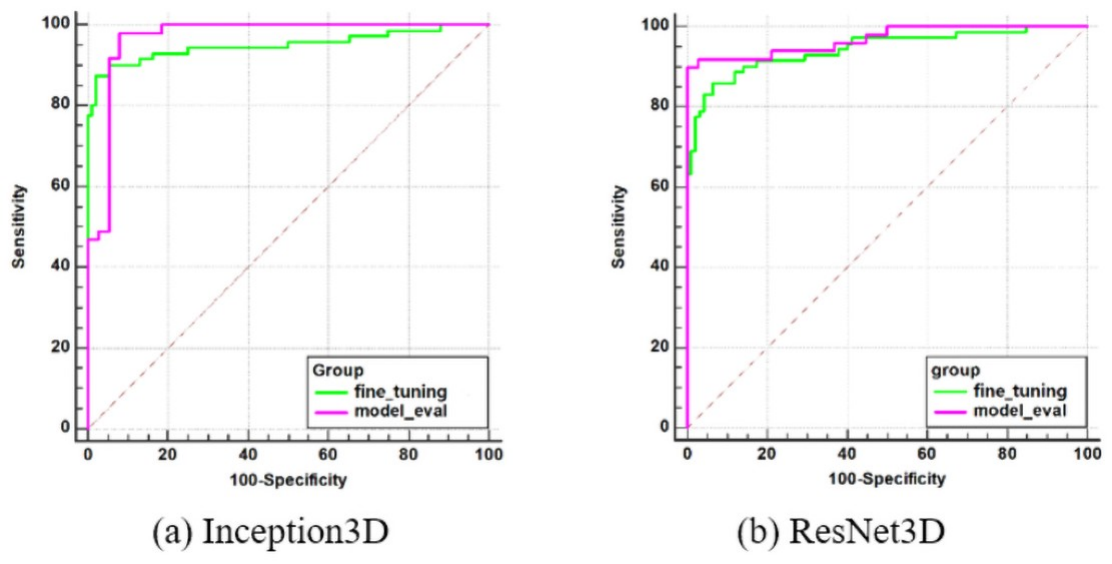
ROC curve comparison between the external validation after retraining and the model evaluation. The ‘*fine*_*tuning*’ curve indicates the external validation after retraining and ‘*model*_*eval*’ indicates the model evaluation.

As summarized in Table 5, when the external validation was performed, the classification performance was improved compared to before retraining. The evaluation performance of the models trained with DAUH was compared with that of models additionally retrained with the residue of the external datasets by independent ROC. Both evaluation performances were within the 95% confidence level (Inception3D: 0.5124, ResNet3D: 0.3247), as shown in Table 6. No significant difference was observed between the two evaluations for each model, and the ROC curves are shown in Fig 10.

**Table 5:** External validation after model retraining.

	Inception3D	ResNet3D
AUC	0.95	0.943
Sensitivity	0.845	0.915
Specificity	0.967	0.783
Accuracy	0.92	0.84

**Table 6:** Comparison of independent ROC curves with the model evaluations and the external validations after retraining.

	Difference between areas	SE	95% CI	P-value
Inception3D	0.0185	0.0283	-0.0369 to 0.0740	0.5124
ResNet3D	0.0251	0.0255	-0.0249 to 0.0751	0.3247

## 4 Discussion

Currently, PET scans are used to diagnose AD by determining the level of amyloid deposition, which is assessed for severity through visual assessment by experts. We also assessed it and obtained Fleiss’ Kappa coefficient to determine it statistically, and the interreader agreement was derived as *κ* = 0.8950. However, this method cannot provide consistent results, as different specialists may interpret images differently. In addition, a doctor’s prior experience can have a significant impact on the reliability of the diagnostic results. Therefore, CNN-based medical image analysis, a deep learning procedure, can produce consistent results and improve confidence in the diagnosis.

Similar to other CNN-based neuroimaging classification studies, our model evaluation of the amyloid positivity classification problem in [^18^F]Florbetaben brain PET yielded an average of approximately 95% accuracy results. In addition, the CNN trained with the single institution dataset demonstrated satisfactory performance when tested with [^18^F]Florbetaben brain PET images obtained from the subjects in the ADNI database. These results support that this CNN model can help diagnose AD by developing a computer-aided detection tool to determine amyloid positivity since these CNN models can recognize the amyloid deposition features of the brain well.

In the external validation, the classification performance of the VGG 3D model was the best with an AUC of 0.945 and an accuracy of 85.3%, but the optimal model may be different if the experimental conditions are different or other model structures are performed. There was also a difference in the classification performance between the model evaluation and the external validation with the Inception3D and ResNet3D models (significance level < 0.05). Thus, a method to overcome these issues is yet to be developed. This improves optimization by fine tuning [28, 29] weights using the learning part or all the layers in Section 3.4.

### 4.1 Open platform

Our CNN-based [^18^F]Florbetaben amyloid brain PET classification studies have a limitation in that only the DAUH dataset and ADNI have been utilized. Therefore, a medical imaging artificial intelligence (AI) research platform that enables doctors with medical knowledge and medical data to perform medical AI research without programming is needed. DEEP:PHI is one such open platform (https://www.deepphi.ai). It is a research platform developed by DEEPNOID, a Korean medical AI startup company that is currently being serviced in the form of a closed beta, and a number of doctors are using this platform to conduct artificial intelligence research.

**Fig 11:**
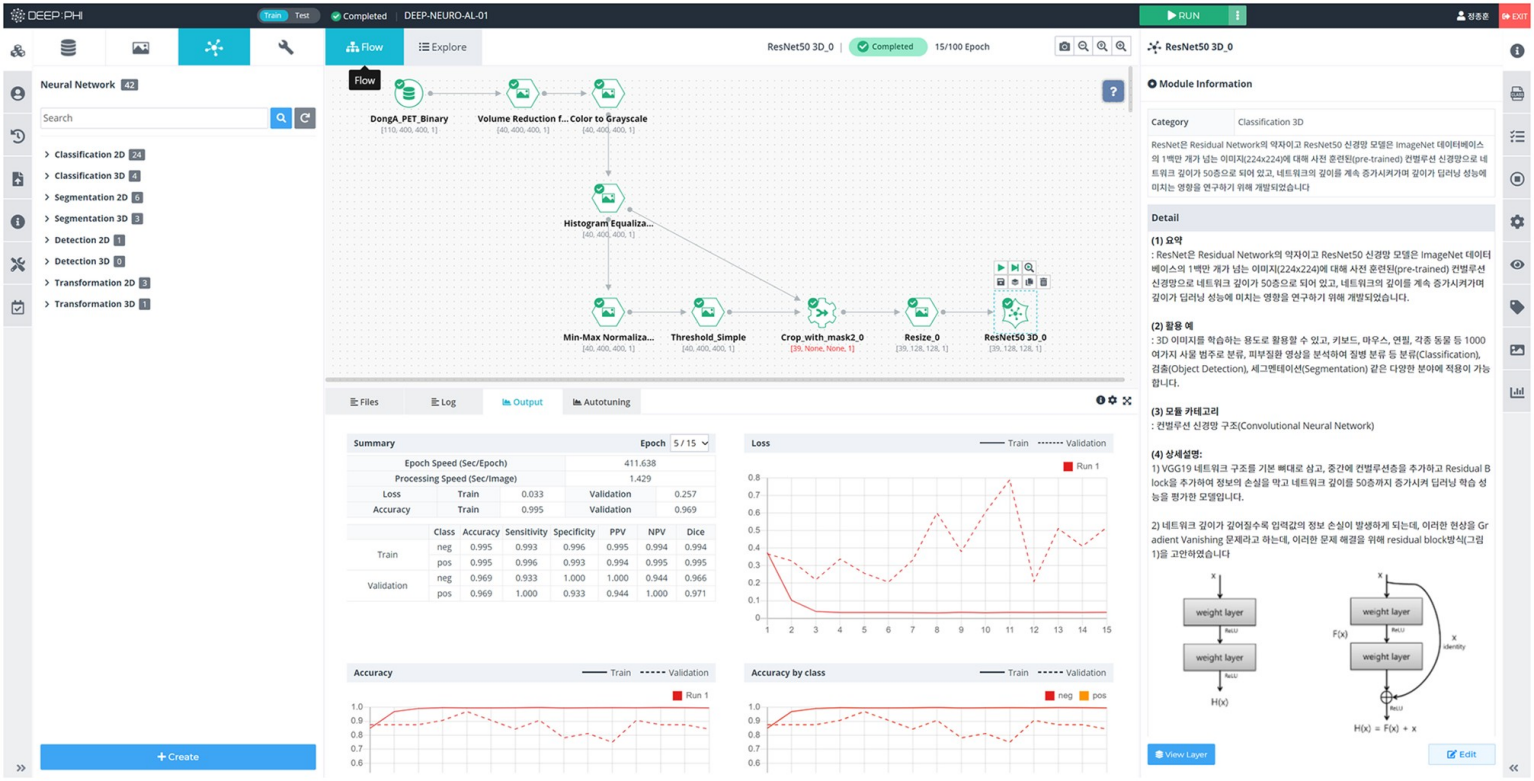
GUI of DEEP:PHI. The image preprocessing, performance, etc. can be seen in the window.

As shown in Fig 11, DEEP:PHI comprises a GUI, and it is possible to perform image preprocessing, neural network model generation, and neural network training results verification within the DEEP:PHI platform from the workflow window. In addition, the server provides a high-end research environment without a GPU and hard disk drive in the local environment. As it operates on the web, the platform allows doctors and developers of various organizations to collect data and perform collaborative research directly on the web. The models from our research can also be uploaded to the DEEP:PHI platform and used for various AI research. If necessary, it is possible to modify and create modules specialized for specific research through the code editor.

## 5 Conclusion

The A*β* classification model was evaluated and external validation was performed with the ADNI dataset. The detailed information on model evaluation and external validation results can be seen in S1 File. The model evaluation results show that the classification performance produces the high accuracy. On the other hand, data from other sources may have differences in quality when compared to the evaluation dataset, which could lead to the poor classification, and preprocessing to minimize these differences is important in external validation. Even though the data have been refined, when the deep learning classification model does not classify tasks well in the external validation step, the model performance can be improved by including other structures or retraining the model. In addition, it is possible to implement an optimal model through various research collaborations using an open platform for medical image AI research.

## Supporting information

S1 File A*β* positive probability using CNN. This is the probability of predicting the label for each CNN model(EXCEL). The label is coded as A*β* positive 1 and A*β* negative 0, and if the probability exceeds 0.5, it is predicted as A*β* positive, and if it is less than 0.5, it is predicted as A*β* negative. One tab is configured for each validation type, and the meaning of the tab name is as follows:
model_eval: model evaluation.external_val: external validation.ext_additional: external validation after the fine tunning.
(XLSX)
